# Supplementary figures and images for: TRIM29 drives ulcerative colitis by disrupting lipid metabolism via lysosomal dysfunction: a multi-omics and experimental study
Source: Front Immunol. 2026 Jan 12;16:1728932. doi: 10.3389/fimmu.2025.1728932 (PMC12832330; doi:10.3389/fimmu.2025.1728932)

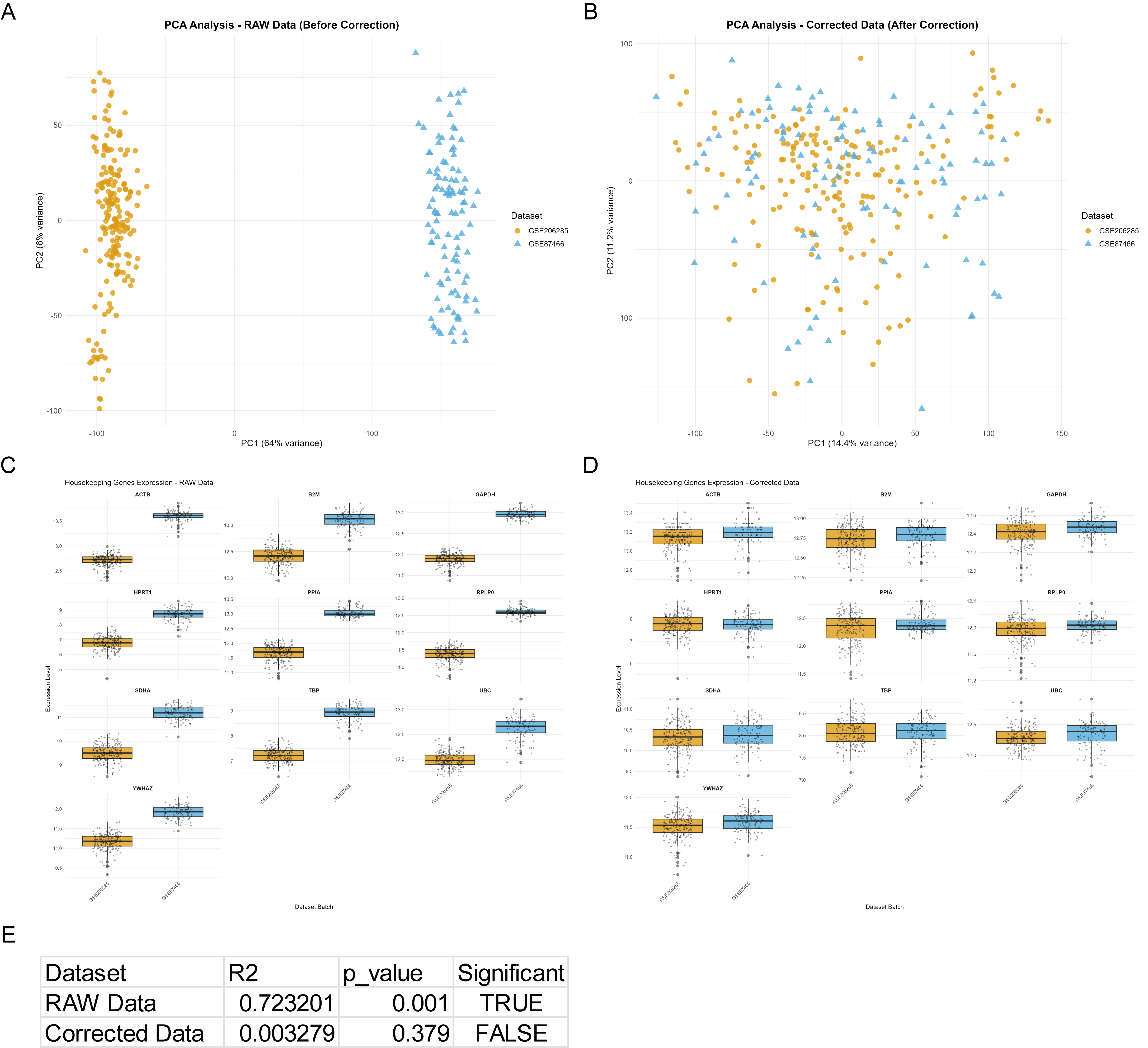

Supplement: Supplementary Figure 1 — Assessment of Batch Effect Correction. (A, B) PCA plots before and after batch effect correction, demonstrating the effectiveness of harmonization. (C, D) Expression distribution of housekeeping genes before and after correction. (E) Statistical significance of batch effects assessed by PERMANOVA. [file Image1.tif]

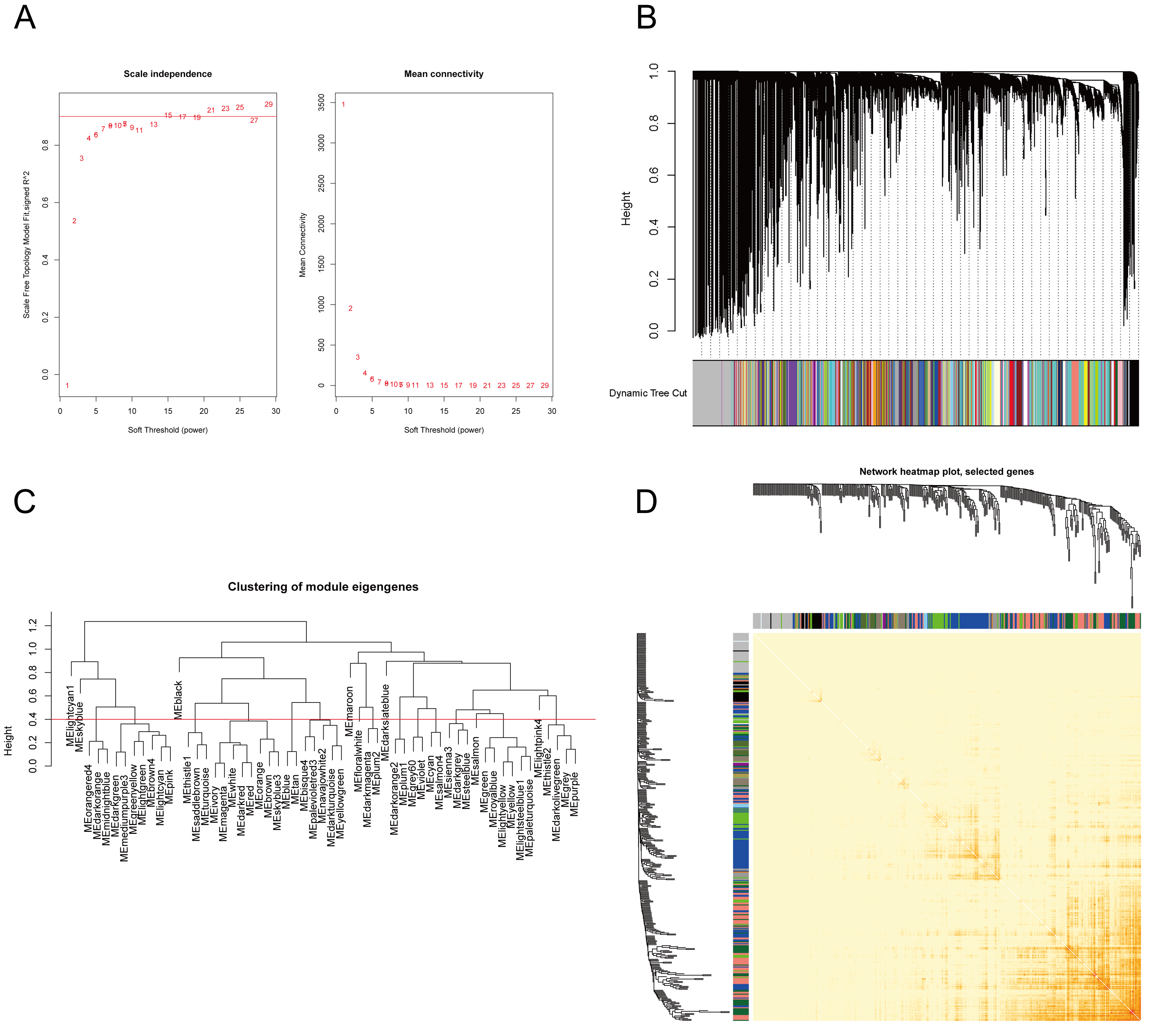

Supplement: Supplementary Figure 2 — WGCNA clustering snapshots. (A) Soft-threshold (left) and mean connectivity (right) plots for power selection. (B) Gene dendrogram with dynamic tree-cut module colors. (C) Module-eigengene clustering dendrogram. (D) Network heatmap of selected genes. [file Image2.tif]

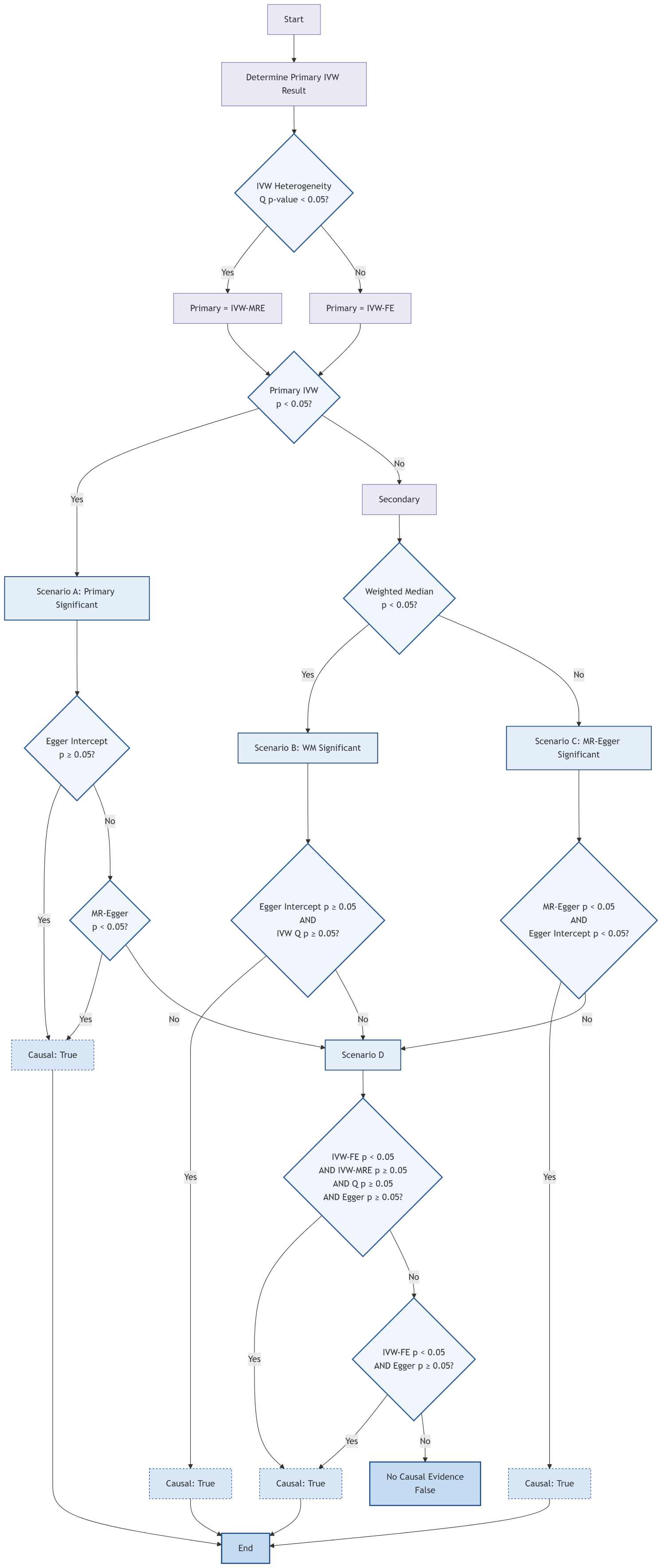

Supplement: Supplementary Figure 3 — Further filtering of significant causal pairs. [file Image3.tif]

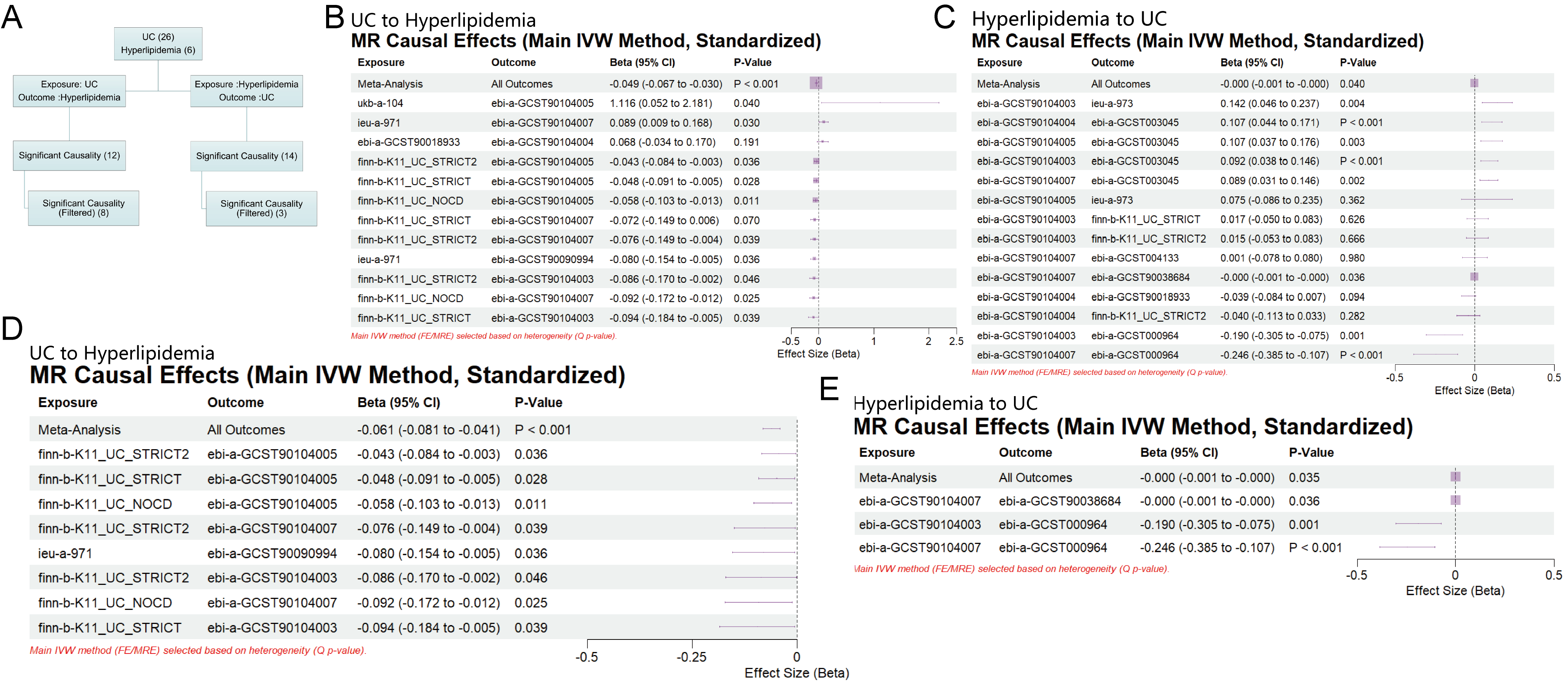

Supplement: Supplementary Figure 4 — Unidirectional causality was observed in both directions across datasets, yet no cohort exhibited bidirectional causality. (A) Flowchart of MR screening results. (B) Significant causal effects of UC on hyperlipidemia. (C) Significant causal effects of hyperlipidemia on UC. (D)Trait pairs with significant UC-to-hyperlipidemia causal effects whose direction agreed with the meta-analysis. (E) Trait pairs with significant hyperlipidemia-to-UC causal effects whose direction agreed with the meta-analysis. Symbol size is inversely proportional to the absolute beta coefficient; larger points correspond to weaker effects. Horizontal lines represent 95% confidence intervals; endpoints indicate upper and lower bounds. [file Image4.tif]

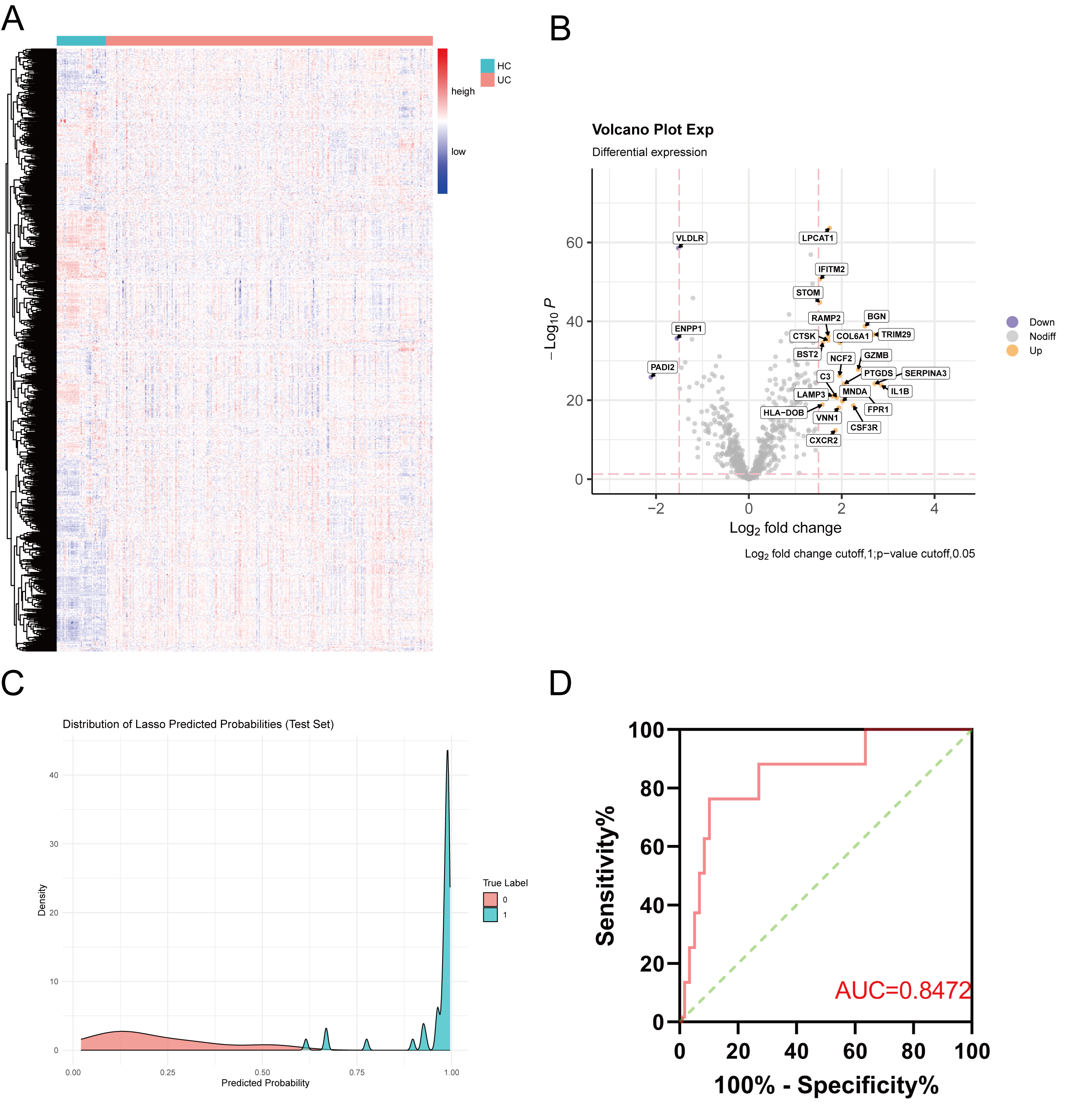

Supplement: Supplementary Figure 5 — Machine learning analysis of the lysosome gene set. (A) Heatmap of differentially expressed lysosome-related genes in the merged dataset. (B) Volcano plot of the lysosome gene set. (C) The distribution of predicted probabilities in the independent test set. The histogram displays the probability scores generated by the LASSO regression model for samples in the held-out test set. (D) ROC curve evaluating the SVM classifier (with 5 feature genes) on the independent test set. [file Image5.tif]

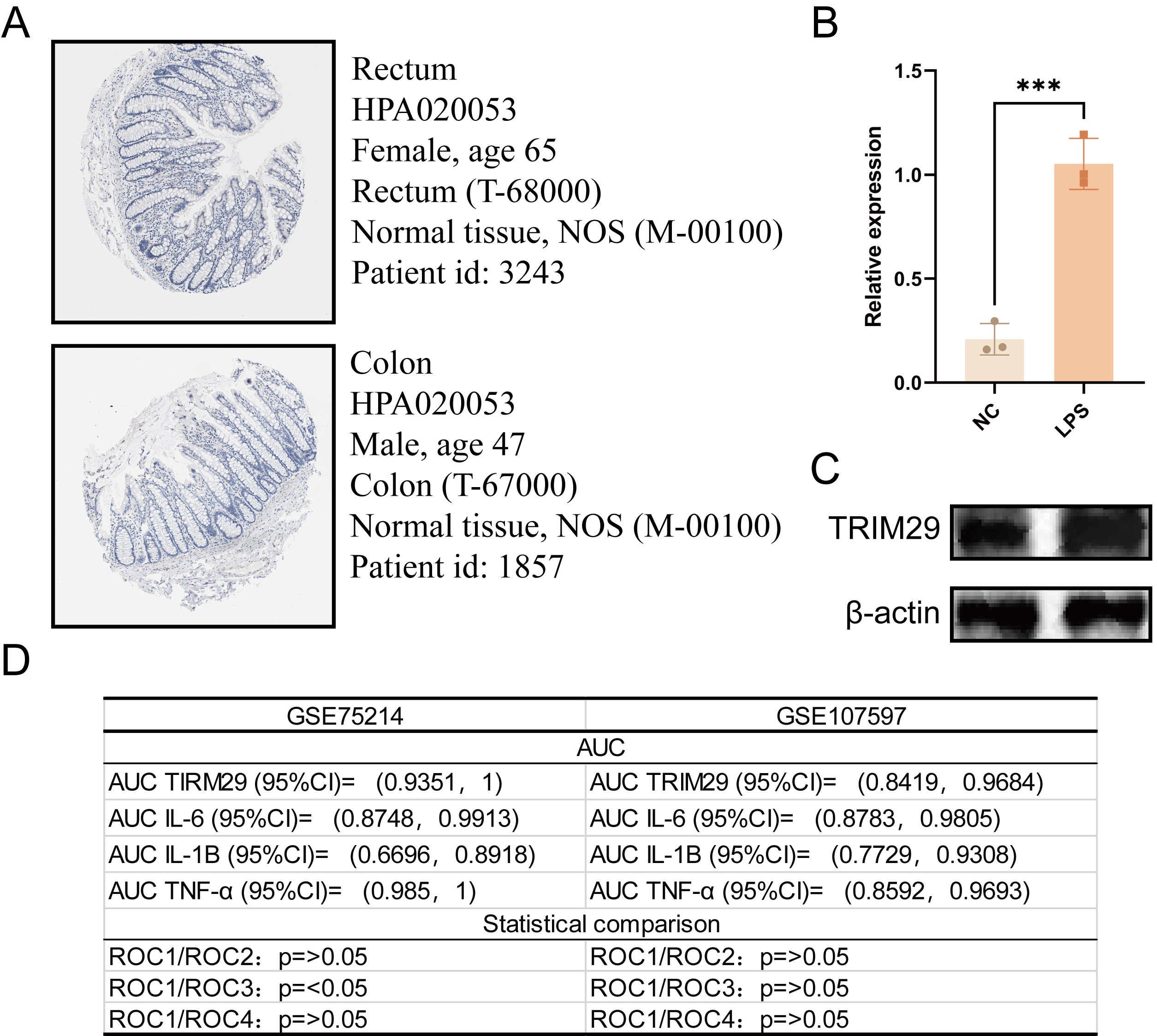

Supplement: Supplementary Figure 6 — TRIM29 as a Biomarker in UC: Expression and Diagnostic Performance. (A) Tissue expression of TRIM29 shown by IHC (Human Protein Atlas). (B, C) Upregulation of TRIM29 at the mRNA (qPCR) and protein (Western blot) levels in LPS-stimulated colonic epithelial cells. (D) Superior diagnostic performance of TRIM29 compared to key inflammatory cytokines in validation datasets. [file Image6.tif]

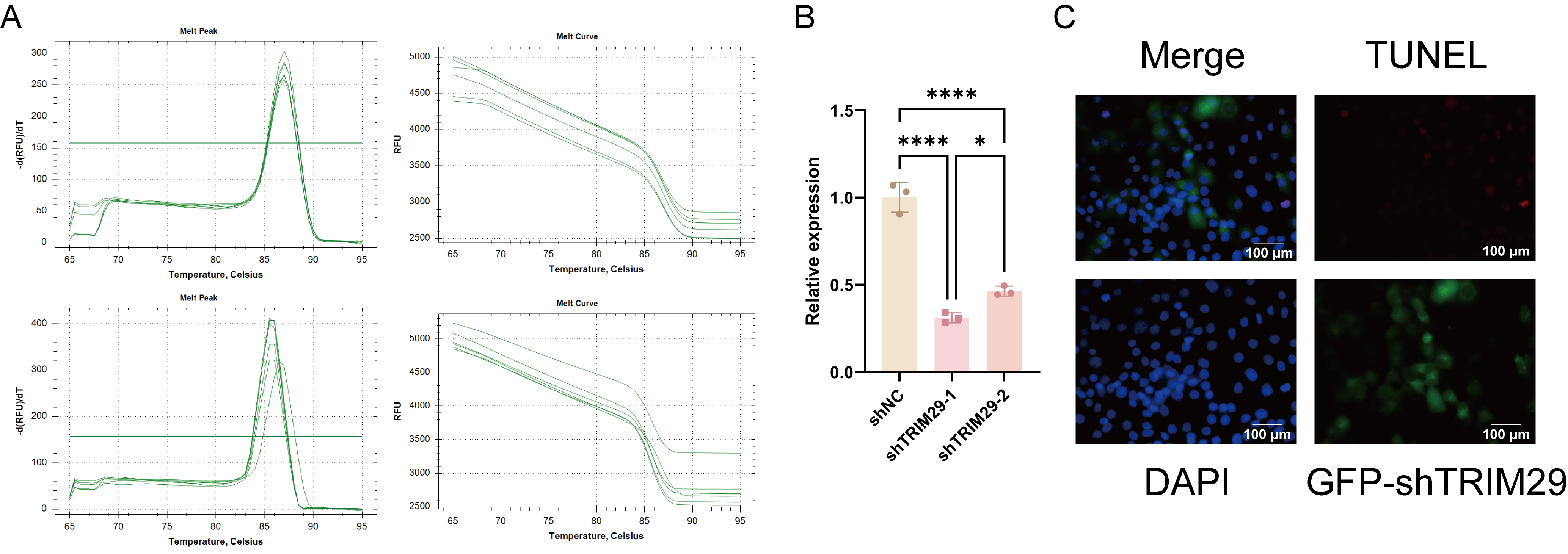

Supplement: Supplementary Figure 7 — Stable CMV-GFP-shTRIM490 NCM460 cell line successfully established. (A) Melt curve and melt peak profiles of ACTB primers (upper panel). (B) Melt curve and melt peak profiles of TRIM29 primers (lower panel). (C) TUNEL apoptotic staining of CMV-GFP-shTRIM29 NCM460 stable cell line. (Red: TUNEL; Green: GFP). All the statistical analysis was performed using unpaired t-tests (for two-group comparisons) or one-way ANOVA (for multiple-group comparisons). (*p < 0.05, **p < 0.01, ***p < 0.001, ****p < 0.0001.). [file Image7.tif]
